# Effect of Needle Insertion Angle on Pain During Labial Infiltration Anesthesia of the Anterior Maxilla: A Randomized Clinical Trial

**DOI:** 10.1002/cre2.70008

**Published:** 2024-09-19

**Authors:** Amirhossein Moaddabi, Tahereh Molania, Alireza Arezoumandi, Sahar Ghaedsharaf, Mariangela Cernera, Roya Nikbakht, Parisa Soltani, Gianrico Spagnuolo, Shirin Shahnaseri

**Affiliations:** ^1^ Department of Oral and Maxillofacial Surgery, School of Dentistry, Dental Research Center Mazandaran University of Medical Sciences Sari Iran; ^2^ Department of Oral Medicine, Dental Research Center, Faculty of Dentistry Mazandaran University of Medical Sciences Sari Iran; ^3^ Students Research Committee, School of Dentistry Mazandaran University of Medical Sciences Sari Iran; ^4^ Private Practice Tehran Iran; ^5^ Department of Neurosciences, Reproductive and Odontostomatological Sciences University of Naples “Federico II” Naples Italy; ^6^ Department of Biostatistics and Epidemiology, Faculty of Health Mazandaran University of Medical Sciences Sari Iran; ^7^ Department of Oral and Maxillofacial Radiology, School of Dentistry, Dental Implants Research Center, Dental Research Institute Isfahan University of Medical Sciences Isfahan Iran; ^8^ Department of Oral and Maxillofacial Surgery, School of Dentistry Meharry Medical College Nashville Tennesse USA

**Keywords:** anesthesia, local, maxilla, needles, pain

## Abstract

**Objectives:**

This study aimed to assess the effect of needle insertion angle on pain during labial infiltration anesthesia in the anterior maxillary region.

**Material and Methods:**

In this parallel‐design randomized clinical trial, participants were randomly assigned to four groups for labial infiltration anesthesia of the anterior maxilla. Local anesthesia was performed with needle orientation parallel to the longitudinal axis of the tooth using a conventional syringe (Syringe‐0), needle at *α* angle with a conventional syringe (Syringe‐α), computer‐controlled local anesthetic delivery (CCLAD) device parallel to the longitudinal axis of the tooth (CCLAD‐0), and CCLAD at *α* angle (CCLAD‐*α*). The heart rate (HR), blood pressure (BP), and respiratory rate (RR) of participants were measured before needle insertion, immediately after needle insertion, and immediately after the injection by a vital signs monitor. The level of pain experienced by participants was quantified using a numerical rating scale (NRS). Data were analyzed by repeated‐measures ANOVA and regression models (*α* = 0.05).

**Results:**

Thirty‐six participants aged from 21 to 60 years, with a mean age of 35.36 years were recruited. The mean pain scores were 7.44, 4.67, 2.89, and 0.67 in groups Syringe‐0, Syringe‐*α*, CCLAD‐0, and CCLAD‐*α*, respectively (*p* < 0.001). Age and sex had no significant effect on pain scores (*p* = 0.914 and *p* = 0.702, respectively). The four groups had no significant difference in vital signs (*p* > 0.05).

**Conclusions:**

Injection at an *α* angle and the application of CCLAD can be used in clinical practice to decrease the pain experienced by participants during labial infiltration anesthesia of the anterior maxilla.

**Trial Registration:**

Iranian Registry of Clinical Trials: IRCT20230719058849N1.

**Abbreviations**
BP: blood pressure
CCLAD: computer‐controlled local anesthetic delivery
CONSORT: Consolidated Standards of Reporting Trials
HR: heart rate
RR: respiratory rate

## 1 Introduction

Despite inter‐individual differences in pain perception, patients usually complain of pain and discomfort after most dental and surgical procedures. Fear of dental anesthetic injections and their associated pain, that is, needle phobia, is a major problem for many dental patients. Evidence shows that one out of every four dental patients is afraid of dental anesthetic injections, and the intensity of fear in one out of 20 patients is so high that leads to avoidance of seeking dental care, which can in turn result in severe consequences (Milgrom et al. 1997; Del Giudice et al. 2021; Cotti et al. 2011; Grund et al. 2015).

Pain is defined as a sensory response to an unpleasant experience related to an actual or potential injury to tissues by internal or external stimuli (Amanat 2004). Pain can be triggered by mechanical impacts or stimuli such as needle insertion or removal. Stress and secondary innervation are among the factors that can affect the efficacy and maintenance of anesthesia (Wang et al. 2014). Also, it has been demonstrated that injection pain negatively impacts patient cooperation, whereas successful anesthesia improves patient cooperation and facilitates the treatment process for dental clinicians (Sharifi et al. 2016). Despite a high success rate, anesthetic injection for infiltration anesthesia in the anterior maxilla is highly unpleasant for patients due to severe pain and pressure felt during needle insertion and the injection of the anesthetic solution (Sharifi et al. 2016; Steele et al. 2013). A previous study on the injection of dental anesthetic agents beneath the mobile mucosa showed a positive correlation between injection pressure and pain severity at the onset of injection. Moreover, the level of anxiety at the onset of injection had a significant positive correlation with injection pain (Kudo 2005; Van Wijk and Makkes 2008).

Infiltration anesthesia is an ideal method for anesthesia of maxillary teeth. It is easy to administer and does not cause lip and tongue anesthesia. The duration of anesthesia is also favorably short (Bajwa et al. 2023). The infiltration anesthesia technique allows for the restoration of teeth in both maxillary quadrants within one session. Thus, stress related to dental procedures can be significantly minimized by reducing the number of treatment sessions.

Several strategies have been proposed to reduce anesthetic injection pain, including changes in needle gauge and design, application of topical anesthesia, changes in anesthetic agent type, use of a modified injection technique, and alteration of the speed of anesthetic release in the tissue, among other approaches (Wang et al. 2014; Moaddabi et al. 2023; Soltani et al. 2023). Considering the adverse effect of pain during anesthetic injection on patient cooperation during a dental procedure, lack of a consensus on one single strategy to minimize pain during infiltration anesthesia of the anterior maxillary region, and the considerable level of pain and pressure experienced by patients in anesthetic injection in this location, this study aimed to assess the effect of needle insertion angle on pain during labial infiltration anesthesia of the anterior maxilla. The null hypothesis was that needle insertion angle would have no significant effect on pain during labial infiltration anesthesia of the anterior maxilla.

## 2 Methods

This study was conducted at the Oral and Maxillofacial Surgery Department of the School of Dentistry, Sari University of Medical Sciences between March and September 2023. The study protocol was approved by the Ethics Committee of the University (IR.MAZUMS.REC.1401.520) and registered in the Iranian Registry of Clinical Trials (registration date: July 31, 2023; registration number: IRCT20230719058849N1).

### 2.1 Trial Design

A parallel‐design randomized clinical trial was conducted in which the four groups received infiltration anesthesia of the anterior maxilla by four different techniques. The results were reported following the Consolidated Standards of Reporting Trials (CONSORT).

### 2.2 Sample Size Calculation

The sample size was calculated to be seven participants per group (a total of 28) according to a study by Razmara, Baghi, and Afkhami (2022), assuming the mean and standard deviation of pain score to be 18.3 and 10.7 in Group 1 and 43.1 and 13.1 in Group 2, with a 95% confidence interval and a study power of 95%. Considering the possibility of dropouts to be 20%, the minimum sample size was increased to 35.

### 2.3 Participants' Selection and Eligibility Criteria

Participants were selected from individuals with hopeless maxillary anterior teeth for whom extraction was required. The inclusion criteria were age between 20 and 60 years (Park et al. 2020), ASA Class I or II general health status (as determined by questioning and assessment of medical records of participants), standardized psychological health status as confirmed by the Persian version of General Health Questionnaire‐28 with confirmed validity and reliability (Taghavi 2002; Rashidi et al. 2018), no intake of medications affecting pain perception, systemic health, no active infection at the injection site, and willingness to participate in the study and sign informed consent forms.

The exclusion criteria were the unwillingness of the individual to participate at any stage of the study and the occurrence of any complications during the injection.

### 2.4 Randomization and Blinding

**Figure 1:**
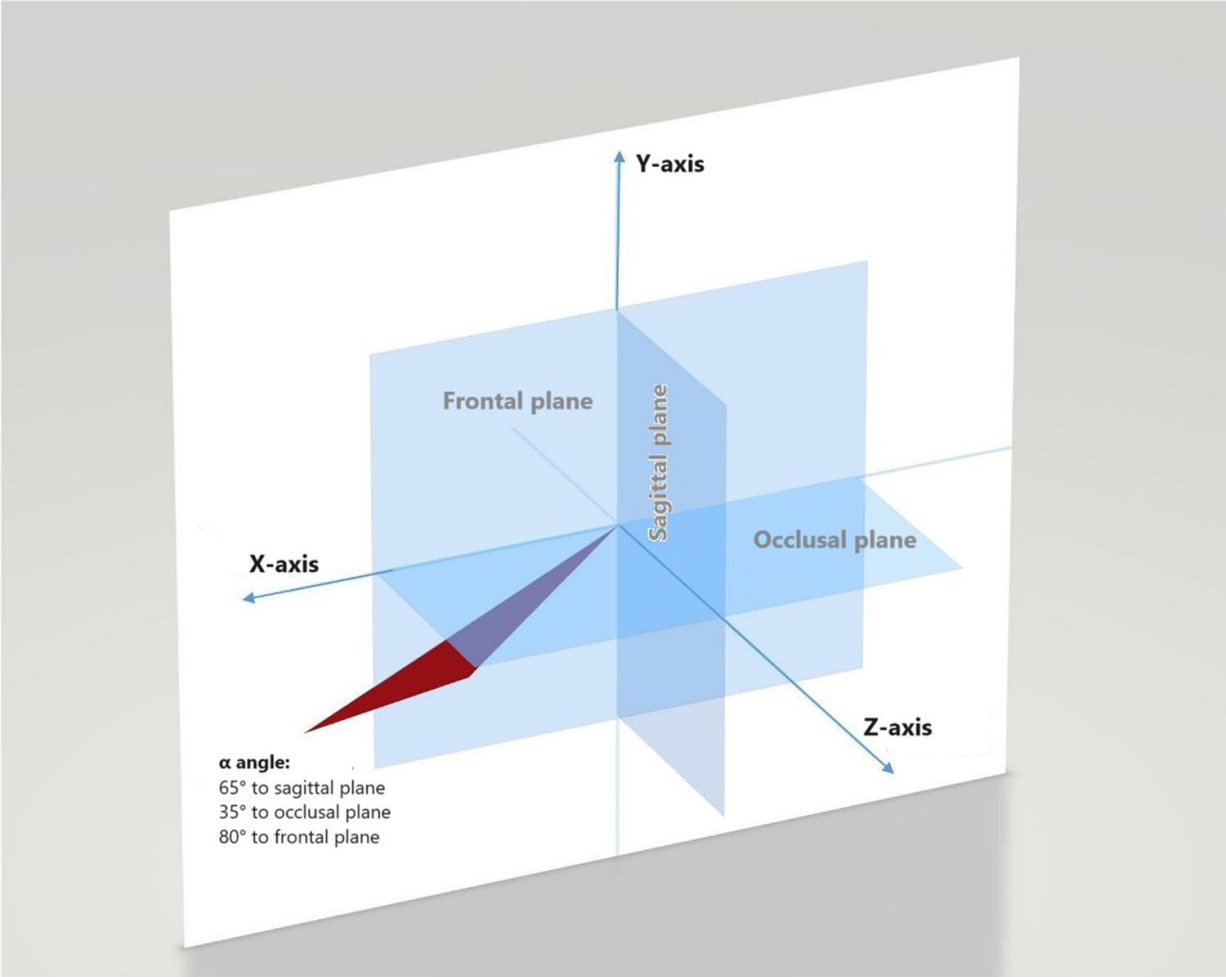
Schematic view of *α* angle.

Participants were enrolled after signing informed consent forms. Block randomization was adopted with four blocks each with a size of 9 using the random allocation software 2.0 (Mahmood Saghaei, Isfahan, Iran). Using this approach, the participants were randomly assigned to four groups for labial infiltration anesthesia of the anterior maxilla with needle orientation parallel to the longitudinal axis of the tooth with a conventional syringe (Syringe‐0), needle at *α* angle with a conventional syringe (Syringe‐*α*), computer‐controlled local anesthetic delivery (CCLAD) device parallel to the longitudinal axis of the tooth (CCLAD‐0), and CCLAD at *α* angle (CCLAD‐*α*). The *α* angle was determined as follows: 65° angle relative to the sagittal plane or longitudinal axis of the tooth, 35° angle relative to the occlusal plane, and 80° angle relative to the frontal plane (Figure 1).

Due to the nature of the study, the operator and participants could not be blinded. However, the statistician was unaware of the allocated group of each participant.

### 2.5 Interventions

The participants were seated on the dental chair and allowed 10 min for their heart rate (HR), respiratory rate (RR), and blood pressure (BP) to return to normal (Daly et al. 2021). Next, the vital signs monitor (CMS 6000, Shenzhen, China) was connected to the patient by an anesthetic technician to monitor the vital signs during the procedure. For adults, normal RR should be 12–20 times/min, normal HR should be 80–100 beats/min, and normal BP should be 120/80 mmHg (Gümüş and Aydinbelge 2020). The vital signs of the participants were recorded at baseline before anesthetic injection, during needle insertion, and immediately after the injection.

**Figure 2:**
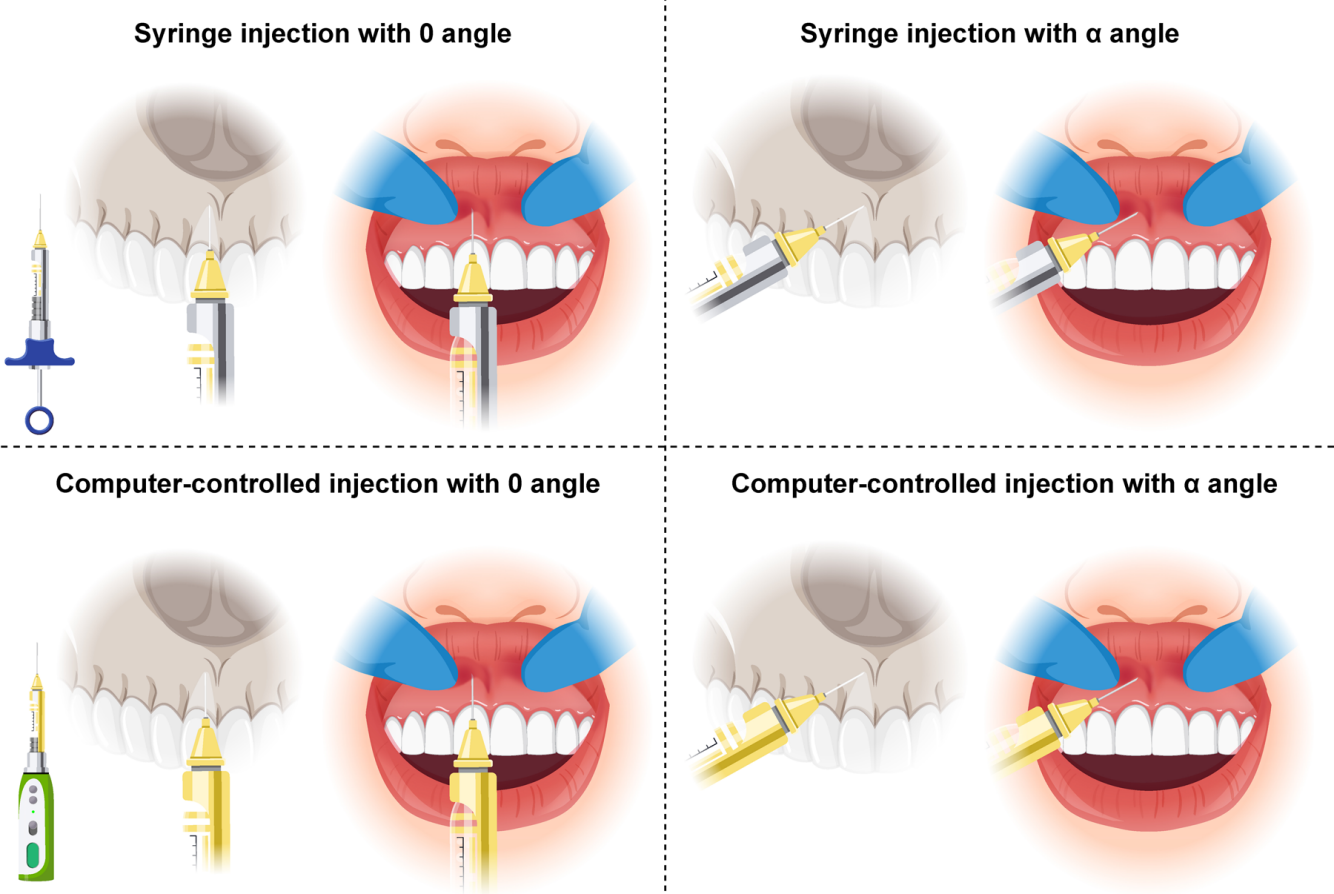
Injection using different syringes and different angles.

The participants were seated in the supine position and requested to open their mouths halfway. The following procedure was then performed for each group of participants:

1) Syringe‐0 group (conventional syringe parallel to the tooth axis): A conventional dental syringe with a short (12‐mm) 30‐gauge needle (Transcodent, Schleswig‐Holstein, Germany) was used for conventional anesthetic injection in this group. The needle was initially inserted parallel to the longitudinal axis of the tooth and proceeded to the apex of the central incisor. Next, 1.2 mL (equal to two‐thirds of the cartridge) was injected close to the apex.
2) Syringe‐*α* group (conventional syringe, needle insertion at *α* angle): The procedure was the same as in the previous group, except that the needle insertion angle was modified. In this group, initially, three anatomical planes—sagittal, occlusal, and frontal—were considered. The needle was then inserted at an *α* angle, which was 65° relative to the sagittal plane or longitudinal axis of the tooth, 35° relative to the occlusal plane, and 80° relative to the frontal plane at the depth of mucobuccal fold of maxillary left central incisor.
3) CCLAD‐0 group (CCLAD device parallel to the longitudinal axis of the tooth): A CCLAD device (ICT Injection GENOSS, Gyeonggi‐do, South Korea) was used in this group, which has three levels for the injection speed (50, 120, and 150 per second). In the present study, the lowest speed was used as instructed by the manufacturer for infiltration anesthesia of the anterior maxilla. A 12‐mm 30‐gauge needle (Transcodent, Schleswig‐Holstein, Germany) was also used. The injection was performed parallel to the longitudinal axis of the tooth and close to the apex, and 1.2 mL of the anesthetic agent was injected (Figure 2).
4) CCLAD‐*α* group (CCLAD device with *α* insertion angle): The process was the same as in the previous group, with the difference that the needle was inserted at an *α* angle, which was 65° relative to the sagittal plane or longitudinal axis of the tooth, 35° relative to the occlusal plane, and 80° relative to the frontal plane, at the depth of mucobuccal fold of maxillary left central incisor (Figure 2).

In all groups, the injections were performed by the same oral and maxillofacial surgeon with over 10 years of clinical experience. The anesthetic drug in all patients was 2% lidocaine with 1:80,000 epinephrine (Darou Pakhsh, Tehran, Iran). The level of pain experienced by the participants was quantified before anesthetic injection, immediately after needle insertion, and after the release of anesthetic agent by using a numerical rating scale (NRS). The participants were asked to select a number from 0 (no pain at all) to 10 (maximum pain imaginable) that best described the level of pain they experienced (Abdelmoniem and Mahmoud 2016).

### 2.6 Primary and Secondary Outcomes

The main objective of this study was to assess the effect of needle insertion angle on pain during labial infiltration anesthesia of the premaxilla. Thus, pain was the primary outcome of this study. BP, HR, and RR were the secondary outcomes.

### 2.7 Interim Analyses and Stopping Guidelines

No interim analyses were performed, and no stopping guidelines were established.

### 2.8 Statistical Analysis

The Shapiro–Wilk test was applied to analyze the normality of data distribution. Considering the normal distribution of systolic and diastolic BP, HR, and RR data, repeated‐measures ANOVA was applied to compare these variables over time within each group. Normal distribution of pain data was also confirmed (*p* > 0.05). Thus, one‐way ANOVA was used to compare the mean pain scores among the four groups, followed by the post hoc Bonferroni test for pairwise comparisons. Repeated‐measures ANOVA was applied to analyze the effect of time, group, and their interaction on vital signs. Linear regression was applied to analyze the correlation between pain scores and demographic variables. Simple linear regression (crude model) was first performed followed by multiple linear regression (adjusted model). The mean age of males and females was compared using an independent paired *t*‐test. All statistical analyses were carried out using SPSS version 22 (SPSS Inc., IL, USA) at 0.05 level of significance.

## 3 Results

### 3.1 Participant Flow

**Figure 3:**
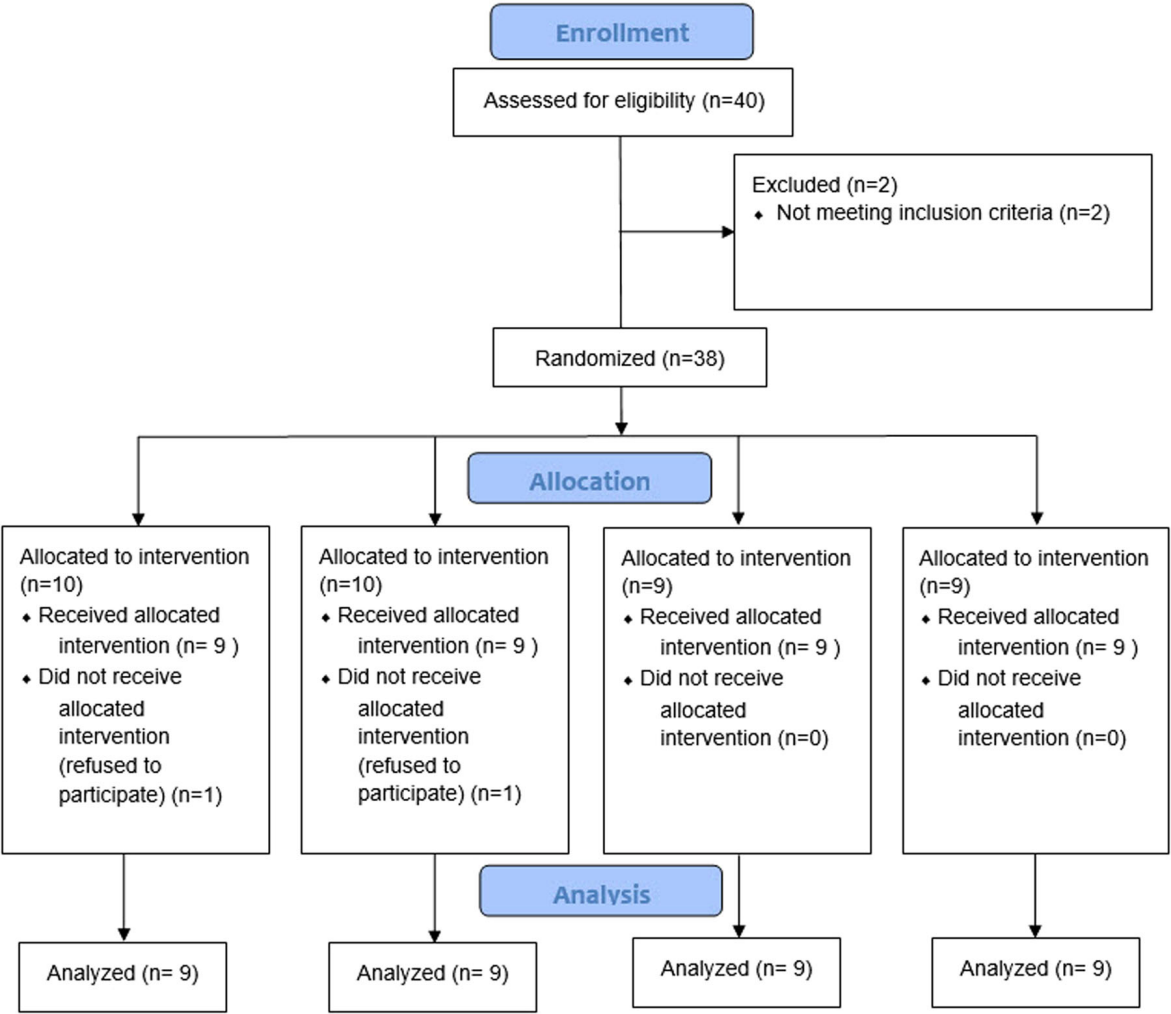
CONSORT flow diagram of patient selection and allocation.

The sample consisted of 36 participants including 22 males (61.1%) and 14 females (38.9%). The mean age of the participants was 35.36 ± 12.87 years (range 21–60 years). The mean age of males and females (32.55 ± 12.45 and 39.79 ± 3.40, respectively) was not significantly different (*p* = 0.101). The mean age was not significantly different among the four groups either (*p* = 0.868). Figure 3 shows the flow diagram of patient selection and allocation.

### 3.2 Subgroup Analyses

#### 3.2.1 Primary Outcome

##### 3.2.1.1 Pain

Table 1 shows the measures of central dispersion for the pain scores in the four groups. One‐way ANOVA showed a significant difference in the mean pain score among the four groups (*p* < 0.05). Pairwise comparisons by the Bonferroni correction (Table 2) showed significant differences between all groups pairwise (*p* < 0.001 in all comparisons).

**Table 1:** Measures of central dispersion for the pain score in the four groups (*n* = 9).

Groups	Mean (SD)	Minimum	Maximum
Syringe‐0	7.44 (0.88)	6	9
Syringe‐*α*	4.67 (0.70)	4	6
CCLAD‐0	2.89 (0.60)	2	4
CCLAD‐*α*	0.67 (0.50)	0	1

**Table 2:** Pairwise comparisons of the groups regarding pain score.

Group (I)	Group (J)	Mean difference	*p* value
Syringe‐0	Syringe‐α	2.77	< 0.001*
	CCLAD‐0	4.55	< 0.001*
	CCLAD‐*α*	6.77	< 0.001*
Syringe‐*α*	CCLAD‐0	1.77	< 0.001*
	CCLAD‐*α*	4.00	< 0.001*
CCLAD‐0	CCLAD‐*α*	2.22	< 0.001*

* Indicates a significant difference.

Linear regression showed that only the effect of the experimental group on pain score was significant (*p* < 0.001). The mean pain score in group Syringe‐0 was 6.77 units higher than in group CCLAD‐*α*. The mean pain score in the Syringe‐0 group was 2.77 units higher than that of the Syringe‐*α* group. The mean pain score in the CCLAD‐0 group was 2.22 units higher than that in the CCLAD‐α group. The effects of sex (*p* = 0.702) and age (*p* = 0.914) on pain scores were not significant. Because the sample size was small compared to the number of variables, each variable was first entered into the model as single (crude model) and then variables with a *p* value of < 0.2 were simultaneously entered into a multiple linear model (adjusted model), which showed a significant difference in pain score among the four groups (Table 3).

**Table 3:** Correlation of pain score with different variables.

Variable	Simple linear regression		Multiple linear regression	
	*t*	*p* value	*t*	*p* value
Gender	0.136	0.881	—	—
Age	−0.13	0.711	—	—
Syringe‐0	6.77	< 0.001*	6.69	< 0.001*
Syringe‐*α*	4.00	< 0.001*	4.04	< 0.001*
CCLAD‐0	2.22	< 0.001*	2.25	< 0.001*
CCLAD‐*α*	Reference	—	—	—
Systolic BP (during)	−0.810	0.171	−0.101	0.570
Diastolic BP (before)	−0.523	0.141	0.070	0.826
Diastolic BP (after)	−0.531	0.127	−0.113	0.727

Abbreviation: BP, blood pressure.

* Indicates a significant correlation.

#### 3.2.2 Secondary Outcomes

##### 3.2.2.1 Diastolic BP

Repeated‐measures ANOVA showed the significant effect of time (*p* = 0.002), the insignificant effect of group (*p* = 0.382), and the insignificant interaction effect of time and group (*p* = 0.486) on diastolic BP (Table 4). In other words, the change in diastolic BP was significant over time within each group. However, the change in the mean diastolic BP was not significantly different among the four groups.

**Table 4:** Comparison of systolic and diastolic BP, RR, and HR before, during, and after the anesthetic injection using repeated‐measures ANOVA.

Variable	Group	Mean ± SD			*p* value (effect size: eta‐squared (*η* ^2^))		
		Before (1)	During (2)	After (3)	Time effect	Group effect	Time* group
Diastolic BP	Syringe‐0	7.22 ± 0.44	7.33 ± 0.50	7.44 ± 0.53	0.002 (0.18)	0.382 (0.09)	0.486 (0.08)
	Syringe‐*α*	7.89 ± 0.60	7.89 ± 0.60	7.89 ± 0.61			
	CCLAD‐0	7.67 ± 0.87	7.78 ± 0.67	8.00 ± 0.71			
	CCLAD‐α	7.67 ± 1.22	7.78 ± 1.09	8.00 ± 1.00			
	Total	7.61 ± 0.84	7.69 ± 0.75	7.83 ± 0.74			
Systolic BP	Syringe‐0	11.33 ± 1.12	12.00 ± 1.00	12.44 ± 1.01	< 0.001 (0.78)	0.072 (0.19)	0.108 (0.15)
	Syringe‐*α*	12.78 ± 0.67	13.11 ± 1.05	13.89 ± 0.78			
	CCLAD‐0	12.67 ± 1.22	12.89 ± 1.27	13.89 ± 1.05			
	CCLAD‐*α*	12.11 ± 1.45	12.22 ± 1.56	13.22 ± 1.64			
	Total	12.22 ± 1.24	12.56 ± 1.27	13.36 ± 1.27			
HR	Syringe‐0	84.56 ± 8.06	87.89 ± 8.10	86.56 ± 8.68	< 0.001 (0.66)	0.217 (0.13)	0.564 (0.07)
	Syringe‐*α*	79.56 ± 3.91	83.89 ± 2.76	81.44 ± 2.96			
	CCLAD‐0	87.78 ± 9.11	91.67 ± 8.82	89.11 ± 8.25			
	CCLAD‐*α*	85.22 ± 9.92	88.44 ± 9.78	85.78 ± 9.38			
	Total	84.28 ± 8.30	87.97 ± 8.02	85.72 ± 7.92			
RR	Syringe‐0	17.33 ± 0.71	18.00 ± 0.87	17.33 ± 0.71	< 0.001 (0.28)	0.739 (0.04)	0.919 (0.03)
	Syringe‐*α*	17.22 ± 0.83	17.89 ± 1.05	17.44 ± 0.53			
	CCLAD‐0	17.11 ± 0.78	17.56 ± 1.01	17.11 ± 0.78			
	CCLAD‐*α*	17.11 ± 0.93	17.89 ± 0.78	17.00 ± 0.87			
	Total	17.19 ± 0.78	17.83 ± 0.91	17.22 ± 0.72			

Abbreviations: BP, blood pressure; HR, heart rate; RR, respiratory rate.

* Indicates a significant correlation.

##### 3.2.2.2 Systolic BP

Repeated‐measures ANOVA showed the significant effect of time (*p* < 0.001), the insignificant effect of group (*p* = 0.072), and the insignificant interaction effect of time and group (*p* = 0.108) on systolic BP (Table 4). In other words, the change in systolic BP was significant over time within each group. However, the change in the mean systolic BP was not significantly different among the four groups. Systolic BP increased over time in all four groups.

##### 3.2.2.3 HR

Repeated‐measures ANOVA showed the significant effect of time (*p* < 0.001), the insignificant effect of group (*p* = 0.217), and the insignificant interaction effect of time and group (*p* = 0.564) on HR (Table 4). In other words, the change in HR was significant over time within each group, whereas the change in the mean HR was not significantly different among the four groups. In all groups, HR increased during the injection and then decreased after the injection.

##### 3.2.2.4 RR

Repeated‐measures ANOVA showed the significant effect of time (*p* < 0.001), the insignificant effect of group (*p* = 0.739), and the insignificant interaction effect of time and group (*p* = 0.919) on RR (Table 4). In other words, the change in RR was significant over time within each group, but the change in the mean RR was not significantly different among the four groups. In all groups, RR increased during the injection and then decreased after the injection.

## 4 Discussion

According to the present study, the pain score was the lowest in the CCLAD‐*α* group, whereas the highest score was reported in the Syringe‐0 group. In the syringe and CCLAD groups, the participants for whom the modified angle (*α*) technique was applied reported lower pain scores. The difference in this regard was significant among the four groups. Thus, the null hypothesis of the study was rejected. A search of the literature revealed no previous studies comparing the effect of different needle insertion angles on the perceived pain by patients during anesthetic injections.

Several approaches have been proposed to reduce the pain of dental anesthetic injections, including the application of deeply anesthetizing topical agents, needleless syringes, modifications of injection technique, behavioral management, and sedation. Clinicians should make the decision in this regard by taking into account all the advantages, disadvantages, potential risks, and protective protocols (Soltani et al. 2023; Daly et al. 2021; Gümüş and Aydinbelge 2020; Putrino et al. 2023; Campus et al. 2021).

CCLAD systems have transformed the way dental professionals administer local anesthetics. These systems offer precise needle placement, consistent drug flow, and increased operator control. Clinical studies consistently demonstrate reduced pain perception and improved patient experience with C‐CLAD injections (França et al. 2022). Dentists and hygienists appreciate the ergonomic advantages that allow them to focus on needle positioning, whereas the device administers the drug at a preprogrammed rate. Available systems include The Wand, Calaject, EZ Flow, and DentaPen. Patient acceptance of CCLAD technology is high, making it a promising advancement in local anesthetic techniques (Janik et al. 2024).

CCLAD systems enhance patient comfort by providing reliable anesthesia delivery. Operators can now concentrate on precise needle insertion, whereas the device ensures consistent drug administration. This reduction in pain perception during injections significantly improves the patient experience. As a result, patients increasingly prefer CCLAD systems for future dental procedures. Dentists and researchers recognize the potential of CCLAD technology, and its adoption represents a positive shift in local anesthetic practices. Several studies have shown that the application of CCLAD systems results in a better patient experience during local anesthesia. For instance, Aggarwal et al. (2018) compared the pain score and anxiety of patients in conventional anesthetic injection with the conventional dental syringe and CCLAD. They reported significantly lower levels of pain and anxiety in the CCLAD group. Additionally, they reported that 64.4% of patients preferred CCLAD over conventional syringes. Their findings are in line with those of the present study, indicating less discomfort in patients while injecting using CCLAD (Aggarwal et al. 2018). In another study, Flisfisch, Woelber, and Walther (2021) reported that pain and anxiety levels during the conventional anesthetic injection technique were three times the rate in the use of CCLAD. They found no significant correlation between injection pain and age or sex, which was similar to the present findings (Flisfisch, Woelber, and Walther 2021). Attia et al. (2022) found no significant difference in needle insertion pain between the conventional and CCLAD techniques. They found no significant difference in pain scores between males and females (Attia et al. 2022). Their results regarding the lack of a significant difference in pain between males and females were similar to the present findings. Based on the findings of a systematic review of the literature by França et al., computerized techniques for anesthesia generally cause less pain and discomfort for patients (França et al. 2022). In 2022, Shetty et al. compared the conventional method and CCLAD in pediatric dental patients and reported significantly lower pain scores in the CCLAD group. Systolic BP was significantly different between the two groups only during injection (Shetty et al. 2022). In the present study, the change in BP was not significantly different among the four groups. They found no significant difference in HR between the groups, which was in line with the present findings. However, RR during and after the injection had a significant positive correlation with the use of CCLAD, compared with the conventional method, which was different from the present results probably due to the different age ranges of patients in the two studies. The Hawthorne effect, that is, the alteration of behavior by the subjects of a study due to their awareness of being observed, may also influence the pain perception and result in higher pain scores reported by the study participants (Partido et al. 2020). One of the reasons associated with less patient discomfort during injection using CCLAD is its ability to deliver the anesthetic solution at a constant speed. In addition, the small gauge of the needle makes the insertion of the needle less painful (Kende et al. 2023).

The pain perceived by patients during anesthetic injection is due to two factors: (I) pain due to the needle insertion into the tissue, and (II) pain due to the pressure of the anesthetic agent on the adjacent tissues (Perry et al. 2015).

The rationale for the modified angle of the injection was to avoid direct distribution of the anesthetic solution into the nasal region, particularly in the area of the nasal aperture. This area with its rich innervation from the maxillary nerve is sensitive to the dispersion of the solution (Karaaslan et al. 2007). The basis of this effect can be similar to the avoidance of subperiosteal injection to avoid the resultant pain. The modified angle allows for decreasing the perceived pain and pressure of the anesthetic in this region.

To the authors' knowledge, this study was the first to assess the angle of needle insertion in the conventional and CCLAD techniques on anesthetic injection pain in the anterior maxilla. One of the limitations of the present study was the inability to blind both the operator and participants. In this study, the investigation focused solely on the pain associated with the injection. However, the long‐term effects of varying needle insertion angles were not addressed. Moreover, we ensured that the groups were matched for age and sex. However, within each group, the distribution of age and sex, along with the sample size, did not permit a meaningful analysis. Additionally, because no previous study has been performed on the topic, the comparison of the findings with those of others was not possible. Further investigations with larger sample sizes could provide additional insights into the impact of injection angle on injection pain. Moreover, dental professionals should be educated on the importance of needle insertion angles and trained in techniques to minimize pain during anesthesia. Tailoring our techniques to individual patient needs ensures a more patient‐centric approach. Whether it is minimizing anxiety or optimizing comfort, the main goal remains to enhance patients' overall dental experience.

## 5 Conclusions

Injection at an *α* angle and the application of CCLAD can be used in clinical practice for decreasing the pain experienced by participants during labial infiltration anesthesia of the anterior maxilla. Age and gender had no significant effect on the perceived pain. Vital signs experienced a significant change during the procedure but had no significant difference among the four groups.

## Author Contributions

Amirhossein Moaddabi supervised the study, participated in the methodology, and wrote the initial draft. Tahereh Molania designed the study, participated in the methodology, and critically revised the original draft. Alireza Arezoumandi participated in the methodology and wrote the initial draft. Sahar Ghaedsharaf helped in designing the study and wrote the initial draft. Mariangela Cernera participated in data analysis and wrote the initial draft. Roya Nikbakht participated in the methodology and wrote the initial draft. Parisa Soltani participated in data analysis and critically revised the original draft. Gianrico Spagnuolo interpreted the data and critically revised the original draft. Shirin Shahnaseri interpreted the data and critically revised the original draft. All authors approved the final manuscript.

## Ethics Statement

The study protocol was approved by the Ethics Committee of the Mazandaran University of Medical Sciences (IR.MAZUMS.REC.1401.520).

## Consent

The objectives of the study were explained to all individuals, and informed consent was obtained from all the participants. The principles of the Declaration of Helsinki were followed.

## Conflicts of Interest

The authors declare no conflicts of interest.

## Data Availability

The data that support the findings of this study are available from the corresponding author upon reasonable request.
